# rePPG: Relighting Photoplethysmography Signal to Video

**DOI:** 10.3390/biomimetics11040230

**Published:** 2026-04-01

**Authors:** Seunghyun Kim, Yeongje Park, Byeongseon An, Eui Chul Lee

**Affiliations:** 1 Department of AI Bigdata, Daejin University, 1007 Hoguk-ro, Pocheon-si 11159, Republic of Korea; kimsh@daejin.ac.kr; 2 Department of AI & Informatics, Graduate School, Sangmyung University, 20 Hongjimun 2-gil, Jongno-gu, Seoul 03016, Republic of Korea; 202533024@sangmyung.kr (Y.P.); 202433029@sangmyung.kr (B.A.); 3 Department of Human-Centered Artificial Intelligence, Sangmyung University, 20 Hongjimun 2-gil, Jongno-gu, Seoul 03016, Republic of Korea

**Keywords:** remote photoplethysmography (rPPG), physiological signal editing, privacy preservation, generative video modeling, biometric security

## Abstract

Remote photoplethysmography (rPPG) extracts physiological signals from facial videos by analyzing subtle skin color variations caused by blood flow. While this technology enables contactless health monitoring, it also raises privacy concerns because facial videos reveal both identity and sensitive biometric information. Existing privacy-preserving techniques, such as blurring or pixelation, degrade visual quality and are unsuitable for practical rPPG applications. This paper presents rePPG, a framework that inserts a desired rPPG signal into facial videos while preserving the original facial appearance. The proposed method disentangles facial appearance and physiological features, enabling replacement of the physiological signal without altering facial identity or visual quality. Skin segmentation restricts modifications to skin regions, and a cycle-consistency mechanism ensures that the injected rPPG signal can be reliably recovered from the generated video. Importantly, the extracted rPPG signals are evaluated against the injected target physiological signals rather than the subject’s original physiological state, ensuring that the evaluation measures signal rewriting accuracy. Experiments on the PURE and UBFC datasets show that rePPG successfully embeds target PPG signals, achieving 1.10 BPM MAE and 95.00% PTE6 on PURE while preserving visual quality (PSNR 24.61 dB, SSIM 0.638). Heart rate metrics are computed using a 5-second temporal window to ensure a consistent evaluation protocol.

## 1. Introduction

Remote photoplethysmography (rPPG) is a technology that extracts photoplethysmography (PPG) signals in a contactless manner by analyzing subtle skin color variations. Recent advances in deep learning have significantly improved the accuracy and robustness of rPPG signal extraction, enabling better handling of real-world challenges such as motion artifacts and illumination changes [1,2,3,4,5]. Physiologically, the cardiac pulse induces periodic blood-volume changes in the dermal microvasculature, which modulate the skin’s wavelength-dependent absorption and reflectance. These pulsatile hemodynamic changes modulate the light absorption and reflectance properties of the skin, producing subtle temporal fluctuations in pixel intensities that can be captured by a standard RGB camera. In particular, periodic increases in blood volume lead to higher light absorption by hemoglobin and corresponding changes in reflected light intensity recorded in skin pixels. As a result, an RGB camera captures quasi-periodic, minute temporal intensity variations in skin pixels, and rPPG algorithms recover the underlying blood-volume pulse from these color fluctuations. Unlike traditional PPG sensor-based methods, rPPG can measure vital signs such as heart rate (BPM) and blood flow variations without physical contact, making it highly suitable for applications in remote healthcare, emotion analysis, health management and fitness tracking systems.

Biomimetics provides a useful lens to interpret rPPG and the proposed rePPG framework. Contact PPG sensors rely on a bio-optical transduction mechanism: pulsatile hemodynamics in superficial vasculature modulate light absorption and reflectance, which is converted into a measurable temporal signal. rPPG can be viewed as a non-contact, camera-based counterpart of this biomimetic sensing principle, as it infers the same physiological dynamics from subtle skin reflectance variations. Building on this shared bio-inspired foundation, rePPG aims to reembeda controllable physiological trace into video by manipulating only the blood-flow–related optical variations while preserving identity-related appearance cues. Furthermore, our model adopts a bio-inspired closed loop verification strategy. Analogous to biological sensing and regulation systems that use feedback to maintain consistent internal states, rePPG incorporates a cycle-consistency mechanism that re-extracts the rPPG signal from the generated video and enforces agreement with the injected target signal. Together with skin-segmentation guidance that restricts manipulation to vascularized skin regions, this feedback-driven constraint promotes physiologically plausible signal rewriting while maintaining visual realism.

Importantly, the observed facial video can be viewed as a mixture of two factors: (i) external appearance factors that largely determine identity and visual realism (e.g., texture, shading, and non-physiological illumination), and (ii) blood-flow–driven optical variations that appear as subtle temporal color changes in the skin region and carry the rPPG information. By explicitly separating these two components, one can selectively manipulate only the blood-flow–related optical signal while keeping the external appearance unchanged.

With the advancement of rPPG technology, research on extracting biometric information in a non-contact manner has been actively conducted. However, biometric data derived from rPPG signals, such as heart rate (BPM), are highly sensitive personal information. The use of video data in this process raises concerns about privacy protection, as facial images could expose both biometric and identity-related information. Conventional privacy protection methods, such as blurring or pixelation, significantly degrade visual quality, making them impractical for rPPG research and real-world applications. Therefore, a method is required that preserves visual information while modifying the extracted biometric signals. A key insight is that privacy can be enhanced not only by obscuring identity, but also by rewriting the physiological trace itself: if the rPPG-carrying component is controllably altered, the original biometric signature becomes difficult to recover while the face remains visually natural.

It is important to distinguish physiological-signal rewriting from facial de-identification or anonymization. De-identification methods aim to remove or obfuscate identity-related facial cues, whereas the proposed method preserves the facial appearance and instead modifies the physiological signal carried by subtle skin-color variations. Therefore, the scope of privacy addressed in this work is limited to physiological-signal privacy rather than full identity anonymization.

**Figure 1:**
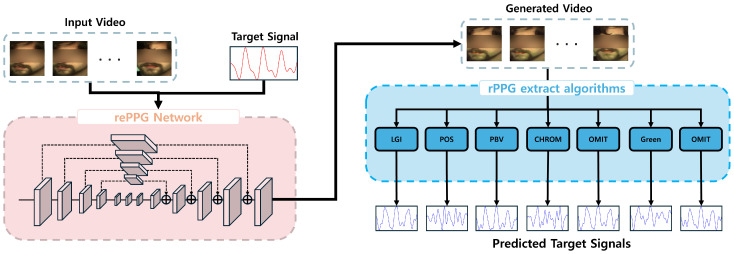
Overview of the proposed rePPG framework. Given an input facial video and a target PPG signal, rePPG generates a relighted video in which the target physiological signal is embedded while preserving the original facial appearance.

This study proposes a novel approach that applies relight techniques to modify rPPG signals while maintaining the visual integrity of the original video, as presented in Figure 1. Our approach extracts rPPG signals from input facial videos, rewrites these signals into new target signals, and regenerates a new facial video that preserves the original facial appearance while reflecting the modified physiological state. In particular, by disentangling blood-flow (physiological) features from external appearance features, our model learns to modulate blood-flow–driven optical information—i.e., the temporal color-change patterns that give rise to rPPG—without altering identity-related visual cues. Importantly, this process is guided by skin segmentation, ensuring that only the skin regions, where the rPPG signals originate, undergo relighting, leaving non-skin areas untouched for enhanced visual realism. Since rPPG is recovered from temporal color variations in skin pixels, the proposed relighting network explicitly intervenes in these variations (e.g., their amplitude/phase patterns over time) during generation so that the reconstructed video yields the desired (rewritten) cardiac dynamics when an rPPG extractor is applied.

The proposed method consists of two core stages:

(1) Facial appearance features and physiological features are first disentangled from the input video, enabling independent processing of each component.
(2) A new target PPG signal is injected into the physiological feature space, and the video is reconstructed using both the modified physiological features and the preserved appearance features. This stage incorporates a cycle consistency mechanism, where the generated video is re-analyzed to verify that the injected physiological signal can be correctly extracted, ensuring the consistency of the relighting process.

Through this framework, we present a practical and privacy-preserving approach for secure rPPG-based video analysis. The main contributions of this paper are summarized as follows:

- We propose a novel PPG relighting framework that preserves facial identity while modifying physiological signals embedded in facial videos.
- We introduce skin segmentation-guided physiological feature modification, which confines signal relighting strictly to the skin regions.
- We design a cycle-consistent reconstruction process that verifies the accuracy and realism of the rewritten physiological signals.
- Our method balances privacy protection and video quality preservation, addressing the limitations of conventional privacy protection approaches.

This study contributes to privacy-aware physiological-signal rewriting in facial videos and may support safer video sharing and controlled physiological video synthesis in privacy-sensitive rPPG applications. The remainder of this paper is organized as follows: Section 2 reviews related work, Section 3 describes the proposed rePPG framework, Section 4 presents the experimental results, Section 5 discusses the findings and limitations, and Section 6 concludes the paper. The implementation code is available at https://github.com/Sangmyung-University-PrLab/rePPG-Rewriting-Photoplethysmography-Signal-to-Video (accessed on 26 March 2026).

## 2. Related Work

### 2.1. rPPG Extraction Methods

Classical rPPG signal extraction algorithms have been widely studied, including the GREEN method [6], which utilizes the green channel of RGB images where skin color changes related to blood flow are most prominent. The CHROM method [7] improves robustness by combining chrominance signals to cancel out lighting variations. POS [8] further enhances signal quality by dynamically adjusting spatial and temporal projection matrices. PBV [9] focuses on using pulse-based variability to extract cleaner signals, while LGI [10] applies matrix completion techniques to reconstruct rPPG signals under challenging conditions such as head motion. ICA [11] is widely used to separate rPPG signals from noise by decomposing them into statistically independent components. The OMIT method [12] introduces an optimized motion-insensitive technique that selectively filters motion artifacts while preserving the underlying rPPG signal. OMIT can improve signal fidelity even in dynamic environments.

Recent studies have increasingly adopted deep learning models for rPPG estimation, which demonstrate improved robustness under challenging conditions such as motion, illumination variation, and diverse skin tones. Representative approaches include DeepPhys [13], which models appearance and motion changes using a convolutional neural network, and PhysNet [14], which directly learns spatio-temporal representations from facial videos to recover physiological signals. Subsequent work such as TS-CAN [15] further improves signal estimation by integrating temporal shift operations and channel-wise attention mechanisms. More recent models also explore transformer-based architectures for capturing long-range temporal dependencies in physiological signals [16].

These advances are particularly relevant to the threat model considered in this study. Modern rPPG systems increasingly rely on deep learning methods due to their superior robustness and accuracy, enabling reliable physiological signal extraction from facial videos. However, most existing rPPG research has focused on improving signal accuracy and robustness, while largely overlooking the potential privacy risks associated with physiological signal recovery. As rPPG technology becomes more capable, facial videos may unintentionally reveal sensitive biometric information such as heart rate and other physiological traits. This highlights the need for privacy-preserving frameworks such as the proposed rePPG, which modify physiological signals embedded in videos while preserving facial appearance.

### 2.2. rPPG-Based Spoofing Detection

Due to the inherent difficulty of forging rPPG as a physiological signal, rPPG-based biometric spoofing detection techniques have been actively researched in recent years. Unlike traditional visual artifact-based detection methods, rPPG-based approaches offer strong security against replay attacks and high-quality synthetic videos.

Deepfake (DF) detection using physiological signals has emerged as an alternative to traditional visual-based approaches. An et al. [17] proposed an rPPG-based DF detection method by dividing the face into five regions and using the neck as the ground truth. By computing the Euclidean distance between the rPPG signals of the facial regions and the neck, they achieved AUC scores of 91.2% and 99.7% on two DF datasets, demonstrating that rPPG signal consistency can serve as an effective biometric for detecting synthetic media.

Various rPPG-based methods have been explored to counteract such attacks, yet many remain vulnerable to replay attacks. Kim et al. [18] proposed an rPPG-based face recognition spoofing detection technique that reduces reliance on specific datasets and enhances resilience against high-quality replay attacks. Without requiring additional hardware, the method employs an RGB camera to extract time-series and frequency-domain rPPG features, achieving a spoof detection accuracy of 99.7424%. Additionally, it validated robustness against novel attack scenarios, such as cut-off attacks targeting the jaw and cheek regions.

However, they also indicate that if these signals remain unaltered, personal information may be exposed, posing privacy risks. Therefore, this study aims to develop a method for manipulating biometric signals in videos to enhance privacy protection and generate more secure visual content.

### 2.3. Privacy-Preserving rPPG Concealment

It has been widely applied in emotion recognition, health monitoring, and biometric authentication. However, this technology inevitably exposes both physiological data and facial identity information, raising serious privacy concerns. Refs. [19,20,21] to address these risks, recent studies have explored various techniques to conceal or modify rPPG signals while preserving their usability.

A representative approach is Privacy-Phys [22], which employs a pre-trained 3D convolutional neural network to modify rPPG signals from facial videos, preventing malicious extraction in scenarios like video calls. However, it performs relighting only at a fixed BPM (e.g., 120), raising concerns about its generalizability to diverse physiological states.

Another method, PulseEdit [23], replaces or removes embedded rPPG signals while maintaining visual fidelity. Similar to Privacy-Phys, it has only been validated on limited BPM settings, and its effectiveness across broader conditions remains uncertain.

Data transformation techniques [22], such as pixel shuffling or facial blurring, help obscure identity while preserving rPPG quality. However, these methods often depend on manually tuned thresholds that vary with environment, limiting scalability and motivating recent shifts toward model-based approaches.

Furthermore, a de-identification framework [24] has been proposed to remove facial identity information from videos while maintaining the quality of rPPG signals. This framework leverages pre-trained face recognition and rPPG prediction models to learn a transformation that preserves physiological utility while ensuring facial anonymity.

Existing methods mainly hide or alter rPPG signals for privacy, but our approach enables direct injection of target physiological signals into videos, allowing controllable and realistic facial video generation for privacy-aware applications.

Unlike previous methods such as Privacy-Phys and PulseEdit, which primarily modify or suppress rPPG signals under limited physiological conditions, the proposed framework enables direct injection of arbitrary target PPG waveforms into facial videos. This capability is achieved through an explicit disentanglement of appearance and physiological features, a ReconNet module that maps target PPG signals into physiological feature representations, and a cycle-consistency mechanism that verifies whether the injected signal can be faithfully re-extracted from the generated video. As a result, the proposed approach allows controllable physiological signal editing while preserving identity-related facial appearance, providing a more flexible and interpretable mechanism for privacy-aware biometric video processing.

## 3. Methodology

Recent advances in remote photoplethysmography (rPPG) have demonstrated that facial videos contain rich physiological information, enabling the extraction of blood volume pulse signals directly from facial skin regions. However, these physiological signals are often entangled with appearance-related factors, such as facial shape, texture, and illumination conditions. This entanglement makes it difficult to explicitly control the physiological signals in facial videos, which limits the flexibility of rPPG-based video synthesis.

**Figure 2:**
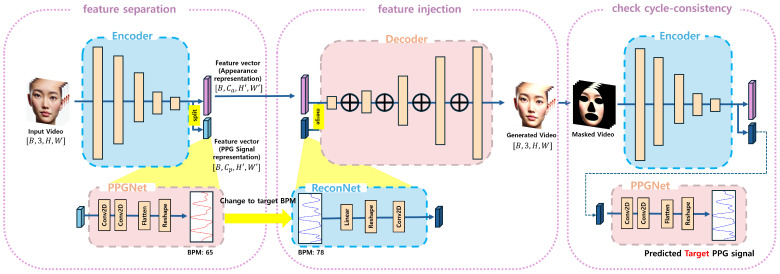
rPPG injection and prediction using rePPG. The rePPG framework is designed to enable the Hourglass network to inject an rPPG signal into a video. It takes an input video and a target PPG signal to generate a video where the target PPG signal is embedded. Subsequently, various rPPG extraction algorithms are applied to the generated video to extract the rPPG signal.

To address this challenge, we propose a two-stage framework designed to (1) learn a disentangled representation that separates appearance features and physiological features and (2) reconstruct facial videos while allowing explicit injection of a target PPG signal into the generated video. This enables controllable editing of physiological signals while maintaining the original facial appearance. We adopt an hourglass-based encoder–decoder architecture [25], which has been widely used for the extraction of structured features in vision tasks. The hourglass design enables multi-scale feature aggregation, allowing effective disentanglement of appearance and physiological features while preserving spatial consistency. The physiological feature maps capture global temporal patterns effectively due to the hourglass encoder’s large receptive field and multi-scale aggregation, which is essential for modeling periodic signals such as rPPG. Figure 2 represents our overall flow. In the following, we describe each stage in detail.

In our implementation, each training sample is represented as a video clip $X\in R^{B\times S\times C\times H\times W}$, where *B* is the batch size, *S* is the sequence length, C=3 denotes the RGB channels, and H×W is the spatial frame resolution. Each sample consists of S=150 consecutive frames generated using a sliding window with step size 30. The overall implementation details of the encoder, PPGNet, and ReconNet are summarized in Table 1.

### 3.1. Step 1: Appearance–PPG Feature Separation and rPPG Signal Extraction

The first stage focuses on disentangling appearance features and physiological features from the input facial video. Specifically, our goal is to decompose the video into:

- an appearance feature map, capturing facial structure, texture, and illumination;
- a PPG-related feature map, capturing subtle skin color changes caused by blood flow.

To achieve this, we use a shared encoder that processes the input video and outputs the two feature maps. The appearance feature map is directly passed to the decoder, preserving appearance information for later reconstruction. The PPG-related feature map is passed to a lightweight PPGNet, which predicts the corresponding rPPG signal. The PPGNet is trained using a supervised loss, comparing the predicted rPPG signal with the ground truth contact PPG (cPPG) signal obtained from a reference device. In addition to the task of simply predicting cPPG signals in PPGNet, we also train a separate model, ReconNet, to reconstruct PPG-related feature maps from input PPG signals. This allows the model to generate appropriate feature maps for target PPG signals in the second step. Both PPGNet and ReconNet consist of simple convolutional layers and MLPs. In this process, the decoder, which takes the feature maps generated from the encoder as input, learns to reconstruct the original input video.

This process forces the encoder to learn a disentangled latent space, where appearance and physiological information are explicitly separated. The PPG-related feature map is optimized to retain only blood flow-related information, minimizing irrelevant appearance content. This separation forms the basis for the controllable video generation process in the next stage. To ensure stable reconstruction of facial appearance, the decoder is first pre-trained prior to the disentanglement stage. This pretraining serves as a foundation upon which the subsequent feature disentanglement and target signal injection processes are applied.

### 3.2. Step 2: Target PPG Injection and Controlled Video Generation

After the training of Step 1 converges, the system can accurately decompose facial videos into appearance and physiological features, as well as reconstruct the original video by combining them. In Step 2, we utilize this learned separation to modify the physiological signal in the reconstructed video. Specifically, we replace the rPPG signal extracted from the input video with a manually designed or predefined target PPG signal, allowing explicit control over the physiological dynamics in the generated video. Target PPG signals were either extracted from real examples or manually synthesized, including signals with 2× or 0.5× the original BPM to simulate different physiological conditions. For manually synthesized signals, sinusoidal waveforms were generated with frequencies corresponding to the desired heart rate. When real signals from other subjects were used as targets, each waveform was normalized by removing its mean value and scaling its amplitude to match the dynamic range of the original signal before injection.

To apply the target PPG signal, the signal is first transformed into a feature map using ReconNet, ensuring that it matches the spatial and channel dimensions of the original physiological feature map produced in Step 1. This target feature map is then combined with the appearance feature map extracted from the input video, and the combined features are passed to the decoder to reconstruct the video. The appearance and rPPG feature maps are concatenated along the channel axis before being passed into the decoder. We found this method preserved more structure and signal fidelity than element-wise addition.

At this point, an additional refinement step is applied to guide the model to focus on physiologically relevant regions of the face. Before feeding the generated video into the encoder for consistency checking, skin segmentation is performed to isolate the facial skin region. Only the skin region is provided to the encoder, ensuring that the physiological feature extraction focuses specifically on areas where blood flow signals are present, such as the cheeks and forehead. This segmentation step helps suppress irrelevant background information and strengthens the association between the injected PPG signal and the facial skin region in the generated video.

Finally, to ensure that the desired target PPG signal is faithfully embedded in the generated video, we perform a self-consistency check. The segmented skin region of the generated video is re-encoded using the same encoder and PPGNet from Step 1, and the newly extracted rPPG signal is directly compared with the injected target PPG signal. This consistency loss serves as a crucial supervisory signal, ensuring that the intended physiological dynamics are correctly embedded in the final video.

This two-stage disentanglement and controlled generation process allows for explicit and localized control over physiological signals, while preserving natural facial appearance. The combination of feature separation, target signal injection, and skin-region-guided encoding makes the framework highly effective for tasks such as physiology-aware video editing and health condition simulation.

### 3.3. Loss Function

To effectively train our two-stage framework, we define a set of loss functions that enforce the correct disentanglement of appearance and physiological features, as well as the accurate reconstruction and modification of physiological signals. Loss functions are applied at different stages of training, ensuring that the model learns to separate, reconstruct, and manipulate rPPG signals while maintaining visual consistency. The overall objective is designed to satisfy four complementary requirements simultaneously: (1) waveform-level physiological consistency, (2) frequency-level heart-rate fidelity, (3) appearance-preserving video reconstruction, and (4) cycle-consistent verification of target-signal embedding. Since no single loss can enforce all of these properties, we combine multiple terms.

#### 3.3.1. Loss for Appearance–PPG Feature Separation

In the first stage, the model learns to disentangle the appearance and physiological features. To achieve this, we define the following loss functions.

##### rPPG Loss ($L_{\mathrm{rPPG}}$)

This loss ensures that the extracted physiological feature map correctly encodes the rPPG signal by minimizing the negative Pearson correlation between the predicted rPPG signal and the ground truth contact PPG (cPPG) signal as in (1):

(1) $$L_{\mathrm{rPPG}}=1-\frac{N\sum xy-\sum x\sum y}{\sqrt{\left(N\sum x^{2}-{\left(\sum x\right)}^{2}\right)\left(N\sum y^{2}-{\left(\sum y\right)}^{2}\right)}}.\;$$

The weighting factor $\alpha_{1}$ is scheduled to decrease exponentially over training epochs:

(2) $$\alpha_{1}=0.1\times 0.5^{\frac{\mathrm{epoch}}{\mathrm{threshold}}}.\;$$

Here, *threshold* denotes the total number of training epochs used to normalize the scheduling function.

##### Frequency-Domain Loss ($L_{\mathrm{fre}}+L_{\mathrm{KL}}$)

To preserve physiological signal fidelity in the frequency domain, we apply two loss functions:

- spectral loss ($L_{\mathrm{fre}}$), measures the difference between the normalized power spectra of the predicted rPPG signal and the ground-truth PPG signal as in (3):
- Kullback–Leibler (KL) divergence loss ($L_{\mathrm{KL}}$) [26], which minimizes the distribution difference between the predicted and actual rPPG signals as in (4):

(3) $$L_{fre}=\frac{1}{N}\sum_{k}\left|S_{pred}\left(k\right)-S_{gt}\left(k\right)\right|$$

where $S_{pred}$ and $S_{gt}$ denote the normalized power spectra obtained using the Fourier transform.

(4) $$L_{\mathrm{KL}}=\sum P\left(x\right)\log\frac{P(x)}{Q(x)}.$$

The weighting factor $\beta_{1}$ follows an exponential growth schedule as in (5):

(5) $$\beta_{1}=1.0\times 5.0^{\frac{\mathrm{epoch}}{\mathrm{threshold}}}.$$

##### Reconstruction Loss ($L_{\mathrm{Recon}}$)

This loss ensures that the feature maps corresponding to rPPG signals can be accurately reconstructed using ReconNet as in (6):

(6) $$L_{\mathrm{Recon}}=\frac{1}{N}\sum {\left(F_{\mathrm{pred}}-F_{\mathrm{target}}\right)}^{2}.$$

##### Mean Absolute Error (MAE) Loss ($L_{\mathrm{MAE}}$)

To ensure accurate heart rate estimation, we include an MAE loss between the estimated and ground truth BPM as in (7):

(7) $$L_{\mathrm{MAE}}=\frac{1}{N}\sum \left|\hat{\mathrm{BPM}}-\mathrm{BPM}\right|.$$

##### Video Similarity Loss ($L_{\mathrm{Similarity}}$)

To maintain the visual fidelity of the reconstructed video, we apply an MSE loss between the generated video and the original input video as in (8):

(8) $$L_{\mathrm{Similarity}}=\frac{1}{N}\sum {\left(I_{\mathrm{gen}}-I_{\mathrm{input}}\right)}^{2}.$$

The total loss function for Step 1 is defined as in (9):

(9) $$\begin{array}{cc} L_{\mathrm{Step}1} & =\alpha_{1}L_{\mathrm{rPPG}-S1}+\beta_{1}\left(L_{\mathrm{fre}-S1}+L_{\mathrm{KL}-S1}\right)\\ \; & \;+\gamma_{1}L_{\mathrm{Recon}-S1}+\eta_{1}L_{\mathrm{MAE}-S1}+\delta_{1}L_{\mathrm{Similarity}-S1}. \end{array}$$

#### 3.3.2. Loss for Target PPG Injection

In the second stage, we modify the rPPG signal in the generated video while preserving its visual appearance. The total loss function for Step 2 consists of two components.

##### Hourglass Video Loss ($L_{\mathrm{Step}2-\mathrm{HG}}$)

This loss ensures that the generated video maintains the correct rPPG signal and appearance as in (10):

(10) $$\begin{array}{cc} L_{\mathrm{Step}2-\mathrm{HG}} & =\alpha_{2}L_{\mathrm{rPPG}-\mathrm{HG}}+\beta_{2}\left(L_{\mathrm{fre}-\mathrm{HG}}+L_{\mathrm{KL}-\mathrm{HG}}\right)\\ \; & \;+\alpha_{2}L_{\mathrm{MAE}-\mathrm{HG}}+\gamma_{2}L_{\mathrm{Similarity}-\mathrm{HG}}+\delta_{2}L_{\mathrm{Recon}-\mathrm{HG}}. \end{array}$$

##### Re-Encoding Consistency Loss ($L_{\mathrm{Step}2-\mathrm{RPPG}}$)

To verify that the injected PPG signal is faithfully embedded, we re-encode the generated video and compare the extracted rPPG signal with the target PPG signal as in (11):

(11) $$\begin{array}{c} L_{\mathrm{Step}2-\mathrm{RPPG}}=\alpha_{2}L_{\mathrm{rPPG}-\mathrm{RPPG}}+\beta_{2}\left(L_{\mathrm{fre}-\mathrm{RPPG}}+L_{\mathrm{KL}-\mathrm{RPPG}}\right)\\ \;+\alpha_{2}L_{\mathrm{MAE}-\mathrm{RPPG}}. \end{array}$$

The final loss function for Step 2 is the sum of both components as in (12):

(12) $$L_{\mathrm{Step}2}=L_{\mathrm{Step}2-\mathrm{HG}}+L_{\mathrm{Step}2-\mathrm{RPPG}}.\;$$

These loss functions ensure that the model effectively disentangles, reconstructs, and modifies physiological signals while preserving the facial appearance in the synthesized videos. The combination of spatial and temporal constraints allows our framework to achieve robust and controllable rPPG-based video synthesis. During Step 2, the encoder and PPGNet weights learned in Step 1 are kept fixed, and only the reconstruction network is optimized under the cycle-consistency constraint.

## 4. Experiments and Results

### 4.1. Datasets

We conduct experiments using two publicly available datasets, PURE and UBFC-rPPG, both widely used in rPPG research [3,27,28,29].

**PURE Dataset:** The PURE dataset [30] consists of facial videos of 10 subjects, recorded under six different head motion scenarios, including steady, talking, and various head movements. Each video is captured at a resolution of 640 × 480 pixels with a frame rate of 30 fps. Ground truth contact PPG signals are provided for each video, making the dataset suitable for supervised learning and evaluation of rPPG algorithms.
**UBFC-rPPG Dataset:** For cross-dataset evaluation, we utilize the UBFC-rPPG dataset [31], which contains facial videos of 42 subjects recorded at a resolution of 640 × 480 pixels and a frame rate of 30 fps. Each video is accompanied by synchronized contact PPG signals. Compared to PURE, UBFC features a wider range of facial appearances, including variations in skin tones and facial shapes. Additionally, all recordings were captured under consistent lighting, providing a complementary testbed for evaluating cross-dataset generalization.

### 4.2. Training Procedure

The training process consists of two distinct steps aligned with our proposed framework. In Step 1, the model is trained for 9 epochs using only the disentanglement objective, where the encoder learns to separate appearance and physiological features. In Step 2, target PPG injection and video reconstruction processes are introduced, and the model is trained for an additional 20 epochs. This staged training strategy helps the model first learn effective feature separation before being tasked with reconstruction and physiological signal embedding. In both Steps 1 and 2, the Adam optimizer with a learning rate of $10^{-3}$ was used.

This study was conducted on a system equipped with an NVIDIA RTX 4080 SUPER GPU, an AMD Ryzen 7 9700X processor, and 32 GB RAM. In terms of computational efficiency, relighting a 10-s video with new physiological signals takes approximately 1.5036 s, demonstrating the practical feasibility of the proposed framework for real-time applications.

### 4.3. Signal-Level Analysis

To quantitatively assess the accuracy of physiological signal embedding, we evaluate the extracted rPPG signals using four commonly used metrics: mean absolute error (MAE), root mean square error (RMSE), peak time error at 6 BPM (PTE6), and Pearson correlation coefficient (PEARSON). MAE and RMSE, both measured in beats per minute (BPM), represent the absolute and squared differences between the extracted and ground truth signals, where lower values indicate higher accuracy. PTE6, expressed as a percentage, measures the proportion of time where the estimated heart rate deviates from the ground truth by less than 6 BPM, with higher values being desirable. PEARSON, a unitless correlation coefficient, quantifies the linear relationship between the extracted and ground truth signals, where values closer to 1.0 indicate a stronger correlation. Unless otherwise stated, the signal-level errors reported in Table 1 and Table 2 are computed between the signal re-extracted from the generated video and the injected target signal, since the objective of rePPG is to rewrite the physiological content of the video rather than to preserve the subject’s original physiology.

#### 4.3.1. Quantitative Evaluation of Embedding Accuracy

To assess whether the target PPG signal was accurately embedded into the generated video, we apply seven well-established rPPG extraction algorithms—GREEN [6], CHROM [7], POS [8], PBV [9], ICA [11], LGI [10], and OMIT [12]—to the generated videos. The extracted rPPG signals are directly compared with the injected target PPG signals, quantitatively verifying the accuracy of physiological signal embedding.

**Intra-Dataset Evaluation:** We evaluate the extracted rPPG signals within the same dataset to assess how well the physiological signals are preserved across different subjects and variations in facial appearances. This evaluation ensures that the target signals are consistently embedded and retrievable under the same dataset conditions. Table 2 presents the quantitative results of various rPPG extraction methods evaluated on the PURE dataset.
**Cross-Dataset Evaluation:** For a broader assessment, we apply the same rePPG model, originally trained on the PURE dataset, to relight the UBFC videos with target PPG signals. We then use the same rPPG extraction algorithms to verify whether the target BPMs are accurately reproduced in the relighted UBFC videos. This analysis demonstrates how well the proposed method relights the target PPG signal to unseen subjects, facial appearances, and environmental conditions. The results in Table 3 indicate the performance variations across different datasets, highlighting the challenges in cross-dataset generalization. Although LGI and OMIT yielded identical rounded summary values in Table 2, the two extractors are based on different projection principles and are not mathematically equivalent. Therefore, these identical table entries should be interpreted as a consequence of aggregated reporting precision rather than as evidence of identical extractor behavior.

#### 4.3.2. Qualitative Assessment of Signal Fidelity

**Figure 3:**
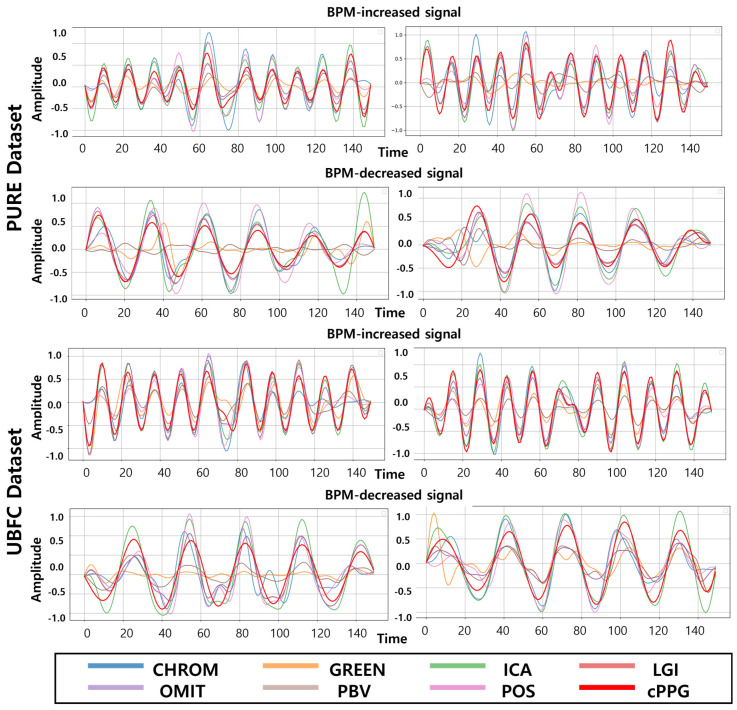
Comparison of extracted rPPG signals. The proposed method successfully embeds target physiological signals while maintaining natural waveform characteristics. For visualization purposes, all signals are normalized.

In addition to numerical comparison, we visually inspect the extracted rPPG waveforms to verify whether the temporal dynamics of the target signals are well maintained. Figure 3 illustrates representative cases comparing the extracted signals from both PURE and UBFC datasets.

### 4.4. Video-Level Analysis

#### 4.4.1. Quantitative Evaluation of Structural and Perceptual Fidelity

To quantitatively measure the visual similarity between the generated and original videos, we compute the average PSNR [32] and SSIM scores across all frames. Higher PSNR and SSIM values indicate better preservation of facial features and structural integrity. We first evaluate the generated frames within the same dataset to assess how well they retain their visual fidelity compared to the original frames. This allows us to analyze the effectiveness of our method in preserving facial details under consistent conditions.

To further examine generalization, we extend our evaluation to unseen datasets, such as UBFC, and compare the results with baseline approaches. This cross-dataset evaluation provides insights into the robustness of our method across different data distributions. The results for the overall quantitative evaluation of visual quality analysis are provided in Table 4.

#### 4.4.2. Qualitative Assessment Through Comparative Visual Analysis

**Figure 4:**
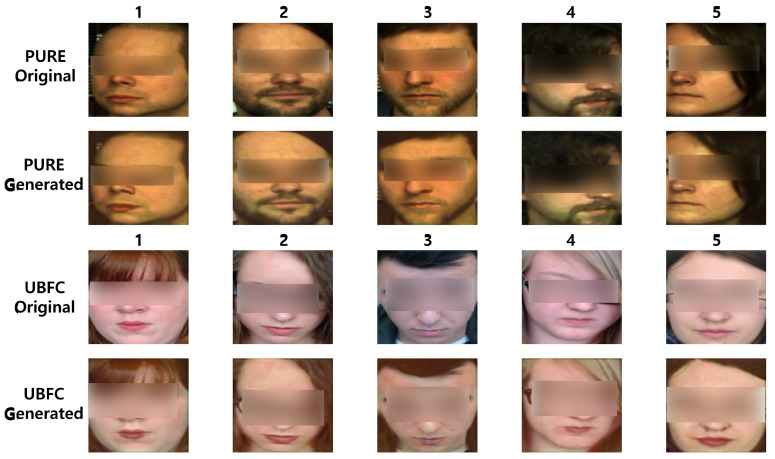
Qualitative comparison of generated frames and ground-truth frames. Our method maintains higher visual fidelity compared to baseline methods.

To visually assess the preservation of facial appearance, we compare sample frames from the generated videos against the original frames. Figure 4 presents a qualitative comparison.

### 4.5. Evaluation of Fixed Target-Signal Embedding

To further validate the accuracy of the proposed rePPG framework in controlled physiological signal manipulation, we conducted an additional experiment focusing exclusively on traditional rPPG extraction algorithms. In this setting, all input videos from the PURE dataset were relighted using a fixed target heart rate of 120 BPM. The relighted videos were then processed using classical rPPG methods to measure how closely the extracted signals match the injected target.

Across all methods, the rePPG-generated videos preserved the target physiological dynamics with perfect accuracy. As shown in Table 5, the mean absolute error (MAE) between the extracted BPM and the injected 120 BPM signal was 0.00 for all classical rPPG algorithms. This confirms that the relighted physiological signals were not only visually seamless, but also faithfully encoded at the temporal level regardless of the extraction method used.

**Figure 5:**
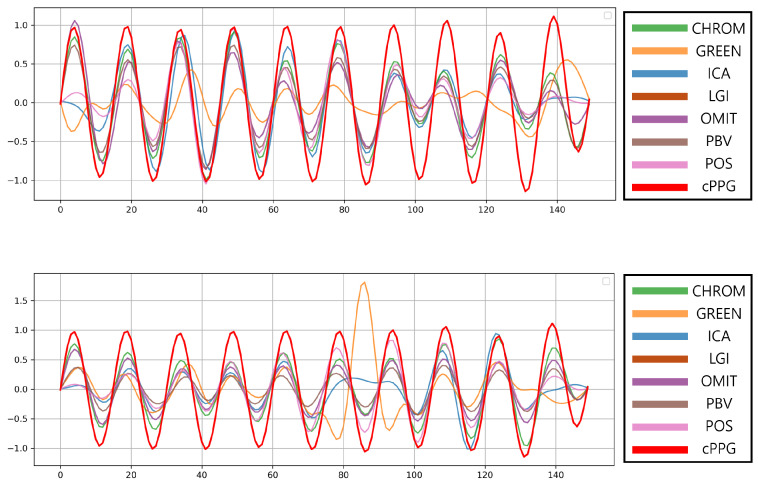
Waveform comparison at 120 BPM using classical rPPG algorithms. Most methods accurately follow the injected physiological rhythm, demonstrating that the relighted videos preserve the intended temporal dynamics.

To further evaluate whether the relighted videos preserve the temporal characteristics of the injected physiological dynamics, we examined the extracted rPPG waveforms when a fixed target heart rate of 120 BPM was applied to all PURE videos. As shown in Figure 5, most classical algorithms (e.g., CHROM, ICA, LGI, POS, PBV, OMIT) closely follow the injected waveform, reflecting that the target periodicity is accurately embedded by the proposed rePPG framework. Although some methods such as GREEN exhibit weaker waveform fidelity, this variation stems from algorithmic limitations rather than the relighting process itself, as confirmed by the MAE of 0.00 BPM in Table 5.

## 5. Discussion

### 5.1. Signal Quality Analysis

This study evaluated the performance of various rPPG extraction methods in intra-dataset and cross-dataset settings, highlighting significant performance differences due to variations in signal modeling, motion robustness, and domain generalizability. In the intra-dataset evaluation, OMIT and LGI outperformed other methods due to their optimized signal decomposition and motion compensation techniques, which effectively reduced motion artifacts and enhanced signal quality. In contrast, ICA, CHROM, and POS exhibited lower accuracy due to their high sensitivity to motion artifacts and illumination changes, as they rely on independent component analysis and color space transformations without explicit motion correction. PBV and GREEN showed the weakest performance due to their simplistic signal extraction, which lacks robust filtering and adaptation to varying physiological and environmental conditions.

In the cross-dataset evaluation, all methods experienced performance degradation, with color-based approaches being particularly affected by environmental and illumination variations. The performance drop in ICA, CHROM, and POS can be attributed to dataset-specific variations in skin reflectance, camera settings, and motion dynamics, which these methods fail to account for. OMIT and LGI demonstrated relatively better generalizability by incorporating motion compensation and adaptive filtering mechanisms, allowing them to maintain higher accuracy across datasets.

### 5.2. Visual Quality Analysis

The results in Table 4 and Figure 4 show that our framework effectively preserves facial appearance while embedding target physiological signals. High PSNR and SSIM values indicate minimal degradation in visual quality after rPPG manipulation. Our method successfully disentangles physiological and appearance features, preventing unwanted distortions. Intra-dataset evaluation (PURE to PURE) confirms this, while cross-dataset evaluation (PURE to UBFC) shows a slight performance drop, likely due to domain shifts such as variations in facial texture and lighting. However, visual fidelity remains robust, as seen in Figure 4. Skin segmentation further enhances realism by ensuring that physiological feature extraction focuses only on relevant regions. This reduces noise from non-facial areas, improving consistency in rPPG embedding. Future work could improve generalization through domain adaptation or perceptual loss functions. Overall, our method provides a strong balance between physiological control and appearance preservation, making it suitable for applications in health monitoring, video editing, and synthetic data generation.

### 5.3. Necessity of Masking

**Figure 6:**
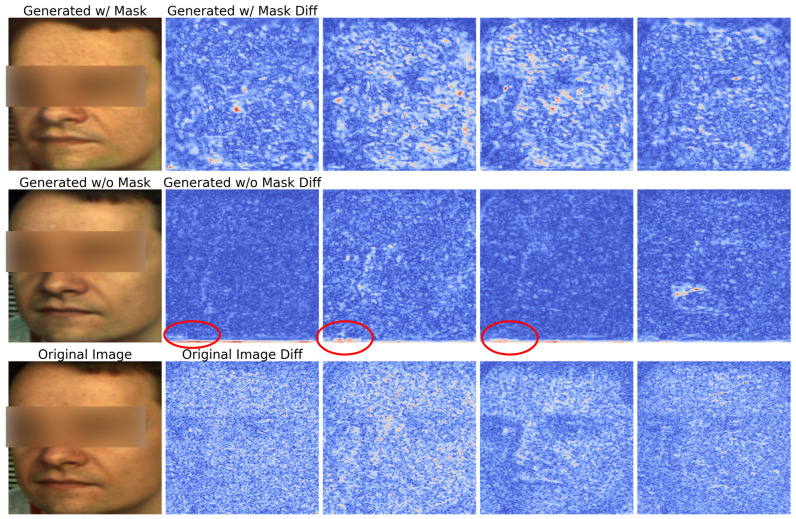
Comparison of video generation results with and without facial masking in the rePPG framework. Without masking (second row), brightness artifacts appear in non-facial regions (highlighted by red circles), indicating deceptive learning. With masking (third row), such artifacts are eliminated, and pixel variations remain confined to the facial area, enabling more reliable rPPG extraction.

The rePPG framework applies masking to retain only facial regions before encoding. We conducted experiments comparing video generation with and without masking, visualizing the 1st, 3rd, 5th, 7th, and 9th frames (Figure 6). Without masking, brightness variations appeared in non-facial regions, suggesting the model embedded the PPG signal into irrelevant areas. With masking, artifacts disappeared and pixel variations remained within the face, ensuring more reliable rPPG extraction.

### 5.4. Applying the Same Signal to a Different Person

**Figure 7:**
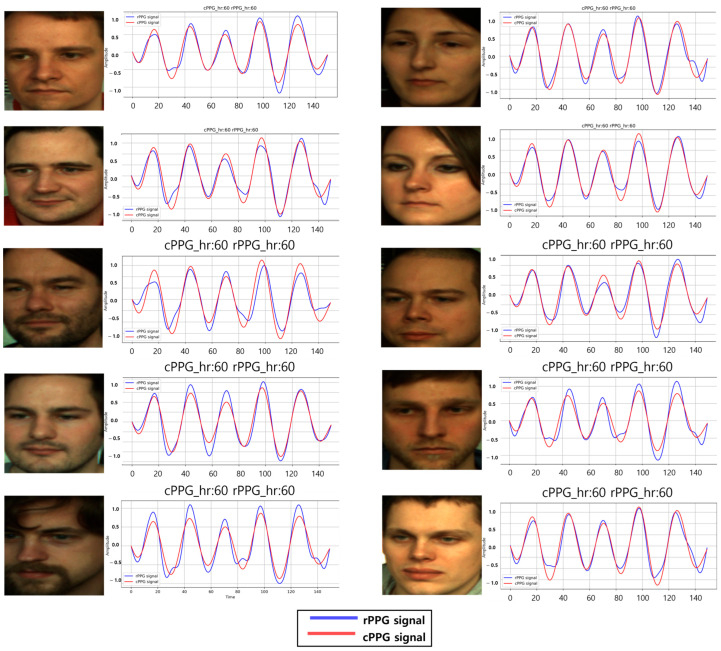
Results of the same manipulated signal extracted from different videos. The blue signal represents the target cPPG signal we injected, while the red signal is the rPPG extracted from the relighted videos using an rPPG method.

We applied the same signal to six individuals to verify consistent BPM across subjects. The signal, extracted using OMIT, was applied uniformly, as shown in Figure 7. All subjects exhibited the same BPM with a correlation of 0.97, demonstrating accurate application.

### 5.5. Limitations and Future Work

Although the proposed framework demonstrates promising results, several limitations remain. First, the skin segmentation module is applied on a per-frame basis to restrict physiological signal manipulation to facial skin regions. However, segmentation errors may introduce minor leakage of non-skin pixels or imperfect masking, which could influence the injected signal distribution. A systematic ablation analysis with quantitative signal-leakage metrics would further clarify this effect. Second, under certain lighting conditions or extreme signal amplitudes, the relighting process may introduce subtle chromatic flicker artifacts. These artifacts may correlate with illumination variations or skin tone differences and should be investigated in more detail. Finally, the datasets used in this study (PURE and UBFC-rPPG) contain a limited number of subjects, which may restrict large-scale generalization analysis. Future work will explore larger and more diverse datasets and investigate domain adaptation strategies and perceptual constraints to further improve robustness and generalization. The visual-quality analysis in Table 3 is currently limited to aggregate PSNR and SSIM summaries. A more detailed characterization across subjects, motion scenarios, and perceptual similarity metrics would further strengthen the evaluation and remains an important direction for future work. In addition, the impact of the generated videos on downstream tasks, such as biometric authentication or physiological monitoring systems, has not been evaluated in this study. Investigating how physiological signal rewriting affects these downstream applications remains an important direction for future work. Finally, because the proposed framework intentionally modifies physiological signals embedded in videos, it may raise potential dual-use concerns in scenarios involving synthetic media or biometric spoofing. Addressing such risks through appropriate safeguards and evaluation protocols will be important for responsible deployment of physiological signal editing technologies.

## 6. Conclusions

In this paper, we proposed rePPG, a novel framework for privacy-preserving rPPG signal relighting and controllable physiological video synthesis. Unlike prior work that only modifies existing rPPG signals, our approach disentangles facial appearance and physiological features, enabling the direct injection of a desired target signal into the generated video while preserving facial identity.

The proposed two-stage process first separates appearance and physiological features to enable accurate rPPG extraction, and then reconstructs the video after injecting the target physiological signal. Skin segmentation guides the relighting process, and a cycle-consistency mechanism ensures that the injected physiological signal can be reliably recovered from the final video, thereby enhancing both privacy and physiological fidelity.

Experiments on the PURE and UBFC datasets demonstrate that our method effectively relights rPPG signals while maintaining high visual quality and strong signal consistency. Nevertheless, minor skin tone shifts remain when embedding highly altered signals, which could be improved by adding a skin tone consistency loss. In addition, some classical rPPG algorithms (e.g., GREEN) still show limited waveform stability due to their inherent sensitivity to illumination and motion, indicating that downstream performance may vary across extractors. These findings suggest that future work should refine the relighting process to improve algorithm-agnostic consistency, reduce model complexity for real-time applications, and further enhance physiological realism so that the generated signals support more comprehensive biometric analysis, such as respiratory rate, heart rate variability, stress, or blood pressure estimation, with stronger cross-dataset generalization.

By directly embedding the temporal morphology of the input rPPG signal into the video, rather than only adjusting scalar measures such as BPM, rePPG provides a controllable bridge between visual content and underlying physiological dynamics. This capability opens up opportunities to apply diverse PPG-based biometric estimation methods in privacy-sensitive scenarios, including metaverse environments or characters with concealed facial information. We believe this framework represents a promising step toward secure, controllable, and physiologically meaningful video synthesis for next-generation remote healthcare systems. 

## Figures and Tables

**Table 1:** Implementation summary of the proposed rePPG framework.

Module	Operation	Output/Role
Input clip	$X\in R^{B\times S\times C\times H\times W}$	S=150 frames, C=3
Pre-convolution	Conv 5×5 + BN + ReLU	16 channels
Hourglass encoder	Multi-scale BasicBlocks	Bottleneck feature extraction
Appearance branch	Bottleneck feature (remaining channels)	Appearance-preserving reconstruction
PPG branch	First 27 bottleneck channels	Physiological feature map
PPGNet	3 Conv layers + FC	10-dimensional latent vector
Temporal head	MLP 10→256→128→1	Frame-wise rPPG value
ReconNet	FC + Conv layers	Reconstructed 27×16×16 feature map
Decoder	Conv layers + output conv	Reconstructed RGB frame

**Table 2:** Performance comparison of rPPG methods on the PURE dataset. Signal-level evaluation on PURE. All error metrics are computed between the rPPG signal re-extracted from the generated video and the injected target signal, not the subject’s original physiological signal. Higher PTE6 and PEARSON values indicate better correlation with the ground truth, while lower MAE and RMSE indicate better accuracy.

Method	MAE ↓	RMSE ↓	PTE6 ↑	PEARSON ↑
OMIT [12]	1.10	5.02	95.00	0.94
LGI [10]	1.30	5.48	94.17	0.94
ICA [11]	2.30	10.56	92.50	0.21
CHROM [7]	3.00	8.90	87.50	0.85
POS [8]	6.45	15.16	77.31	0.43
PBV [9]	26.82	39.63	42.86	0.15
GREEN [6]	42.20	53.08	20.00	0.29

**Table 3:** Cross-dataset evaluation of rPPG methods on the UBFC dataset. Cross-dataset signal-level evaluation on UBFC-rPPG. All reported errors are computed between the signal re-extracted from the generated video and the injected target signal. Higher PTE6 and PEARSON values indicate better correlation with the ground truth, while lower MAE and RMSE indicate better accuracy.

Method	MAE ↓	RMSE ↓	PTE6 ↑	PEARSON ↑
OMIT [12]	3.20	13.77	92.50	0.89
LGI [10]	3.20	13.77	92.50	0.89
ICA [11]	13.40	32.05	75.00	0.37
CHROM [7]	20.60	36.82	55.00	0.64
POS [8]	23.19	40.78	49.58	0.38
PBV [9]	21.18	41.63	59.66	0.24
GREEN [6]	24.10	43.00	55.83	0.37

**Table 4:** Quantitative evaluation of visual quality on the PURE dataset. Higher PSNR and SSIM values indicate better preservation of visual fidelity.

Method	PSNR ↑	SSIM ↑
PURE to PURE	24.61	0.64
PURE to UBFC	20.35	0.63

**Table 5:** Comparison of physiological signal editing methods using classical rPPG extractors. MAE values represent the deviation between the injected target signal and the signal extracted from the relighted videos.

Method	Ours	Privacy-Phys [33]	PulseEdit [23]
POS [8]		0.03	0.03
CHROM [7]	0.00	0.03	0.02
ICA [11]	0.00	0.03	0.03

## Data Availability

The authors do not have permission to redistribute the data.
